# Electronic and electrochemical doping of graphene by surface adsorbates

**DOI:** 10.3762/bjnano.5.195

**Published:** 2014-10-23

**Authors:** Hugo Pinto, Alexander Markevich

**Affiliations:** 1 COMP/Department of Applied Physics, Aalto University School of Sciences, FI-00076 Aalto, Finland; 2 School of Chemistry, University of Nottingham, NG7 2RD Nottingham, UK

**Keywords:** adsorbates, doping, electrochemical, electronic, graphene

## Abstract

Many potential applications of graphene require its precise and controllable doping with charge carriers. Being a two-dimensional material graphene is extremely sensitive to surface adsorbates, so its electronic properties can be effectively modified by deposition of different atoms and molecules. In this paper, we review two mechanisms of graphene doping by surface adsorbates, namely electronic and electrochemical doping. Although, electronic doping has been extensively studied and discussed in the literature, much less attention has been paid to electrochemical doping. This mechanism can, however, explain the doping of graphene by adsorbates for which no charge transfer is expected within the electronic doping model. In addition, electrochemical doping is in the origin of the hysteresis effects often observed in graphene-based field effect transistors when operating in the atmospheric environment.

## Introduction

Graphene is a monolayer of carbon atoms arranged in a honeycomb lattice [1]. With the discovery of graphene a new era of two-dimensional materials for science and technology has started. Due to its remarkable transport, optical and mechanical properties graphene has a great potential for being used in a wide range of applications [2–3]. For instance, high mobility of charge carriers [4] in graphene combined with its high optical transparency [5] and mechanical strength [6] makes graphene a promising material for ultrafast and flexible electronics [7–10]. Graphene is a zero gap semiconductor with its valence and conduction bands touching each other at the corners of the Brillouin zone in the so called Dirac points [11–12]. This implies that, neglecting the effect of thermal excitations, the intrinsic charge carrier concentration in graphene is, in principle, zero. However, for the development of graphene-based electronic devices the presence of charge carriers and the control of their type and concentration are required. Therefore, a lot of research has been devoted to find ways for controllable doping of graphene with electrons and holes. It has been shown that the deposition of different adsorbates, either atoms or molecules, on the graphene surface can result in both n- and p-type doping.

In this paper, we present an overview of two mechanisms of graphene doping by surface adsorbates, namely, electronic and electrochemical doping and consider appropriate atomic and molecular dopants.

## Review

### Mechanisms of doping

Charge carriers, either electrons or holes, can be induced in graphene by the application of an electric field or by chemical doping. The electric field effect doping is usually performed in graphene-based field effect transistors (FET), in which charge carriers are induced by changing the electric potential between graphene and a gate, which can be, for example, a Si^+^/SiO_2_ substrate [1,13]. By varying the gate voltage, V_g_, the type of carriers and their concentration in graphene can be tuned. The sign of the induced carriers is opposite to the sign of the applied gate voltage. A positive V_g_ induces electrons while a negative V_g_ induces holes. It was shown that for graphene on Si^+^/SiO_2_ the concentration of charge carriers induced by this method can be as high as 10^13^ cm^−2^ [1].

Chemical doping involves interactions of graphene with other chemical species [14]. There are two types of chemical doping, surface transfer and substitutional doping. In the latter case doping occurs when some of carbon atoms in the graphene lattice are substituted by other atoms with a different number of valence electrons. This type of doping has been observed for boron, B_s_, and nitrogen, N_s_, substitutional atoms and leads to p- and n-type conductivity, respectively [15–16]. However, the incorporation of foreign atoms into the graphene lattice can result in a significant modification of the electronic structure of graphene. For instance, N_s_-doped graphene behaves like an n-type doped semiconductor but exhibits a low charge carrier mobility [16].

**Figure 1:**
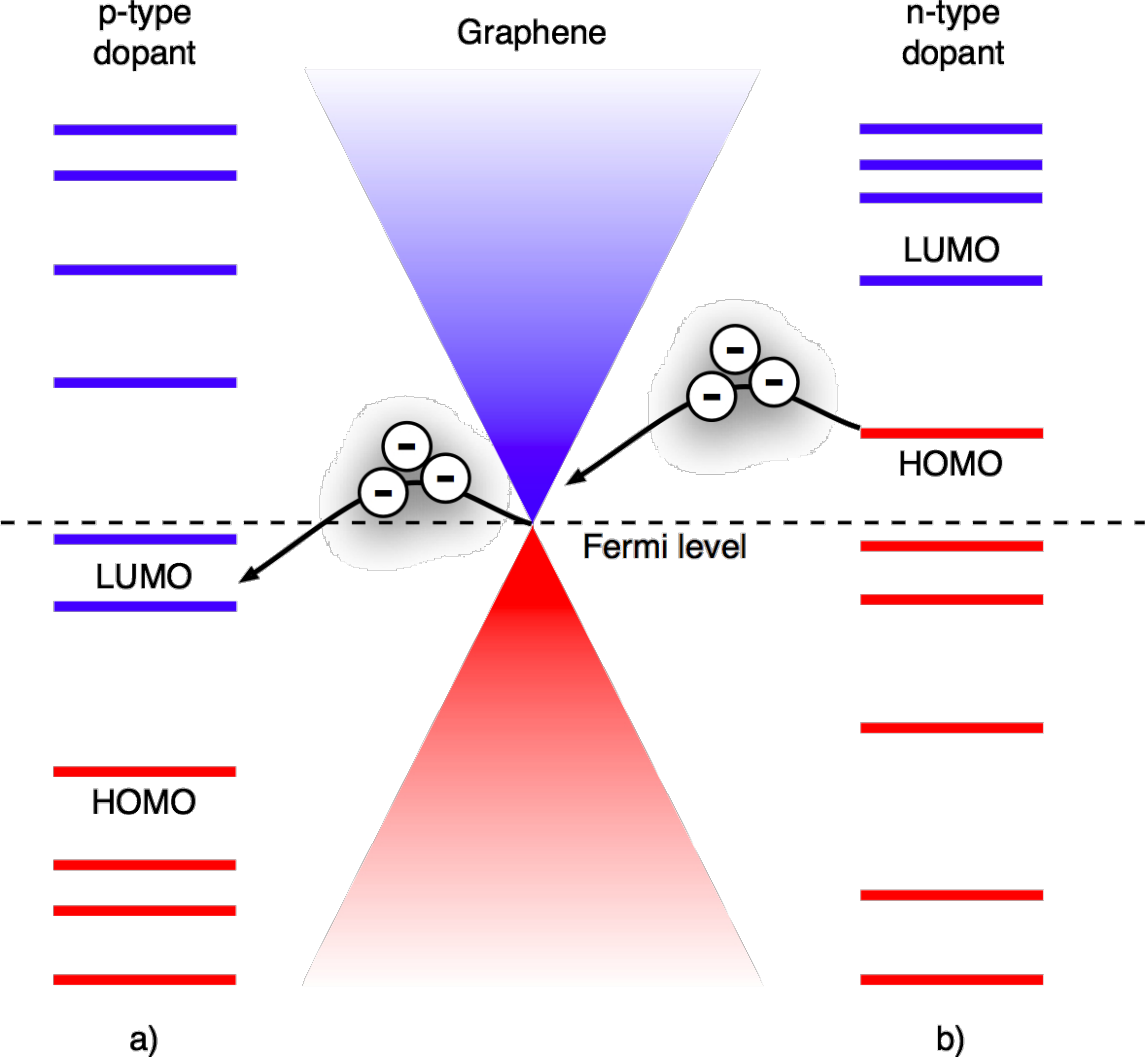
Scheme of the relative position of the highest occupied (HOMO) and lowest unoccupied (LUMO) molecular orbitals of an adsorbate to the Fermi level of graphene for a) p-type and b) n-type dopant.

The surface transfer doping is non-destructive and occurs due to the charge transfer between graphene and surface adsorbates. Two mechanisms of charge transfer doping can be distinguished, electronic and electrochemical doping. The electronic doping is a consequence of the direct charge transfer between graphene and an adsorbate. This requires a difference in electronic chemical potentials at an interface, which is determined by the relative positions of the graphene Fermi level and the highest occupied (HOMO) and lowest unoccupied (LUMO) molecular orbitals of an adsorbate. If the LUMO of the adsorbate lies lower in energy than the Fermi level of graphene, Figure 1a, electrons will flow from graphene to the adsorbate making graphene p-type-doped. Adsorbates with the HOMO lying above the graphene Fermi level, Figure 1b, act as donors and dope graphene n-type. Although surface transfer doping is an effective method to control the concentration of charge carriers in graphene the ionized dopants can act as an additional source of scattering for charge carriers and reduce their mobility.

The electrochemical doping of graphene occurs when certain surface adsorbates participate in electrochemical redox reactions in which graphene plays the role of an electrode. Such reactions occur spontaneously if the total Gibbs free energy is negative and the diffusion and reaction barriers are sufficiently low for the reaction proceed at room temperature. The total Gibbs free energy change is given by Δ*G* + *W* for p-doping and Δ*G* − *W* for n-doping, where Δ*G* is the free energy for the molecular reaction and *W* is the work function of graphene. The work function of graphene is expected to be similar to that of graphite, about 4.5 eV [17–18]. Although Δ*G* can be estimated from the Nernst equation the barrier heights are unknown and it must be assumed that they are sufficiently low for the reaction to occur [19].

Whether a reaction will result in p- or n-type doping of graphene depends on the relative position of the electrochemical redox potential *E*_redox_ to the graphene Fermi level. The *E*_redox_ is the equivalent of the Fermi level of an electrolyte solution. It measures the energy required to add or remove an electron in redox couples and is usually referenced to the standard hydrogen electrode (SHE) [20]. Thus, Δ*G* and *E*_redox_ measure the same property and are related by Δ*G* = −*n*·*F*·*E*_redox_, where *n* and *F* are the number of electrons and the Faraday constant, respectively. Furthermore, while the *E*_redox_ level lies higher (lower) than the Fermi level of graphene (*E*_F_), electrons (holes) induced by a reaction will flow to graphene until equilibrium is reached, *E*_redox_ = *E*_F_, making graphene n- (p-)type doped.

In contrast to electronic doping, which occurs instantaneously, electrochemical doping is a time-dependent process, which is affected by the rate of the reaction and diffusion rates of participating species. Therefore, in the case the reaction or diffusion rates are slower than the rate of change of gate voltage electrochemical doping can lead to hysteresis effects which are often observed in graphene-based FET devices [21–24].

### Electronic dopants

**Figure 2:**
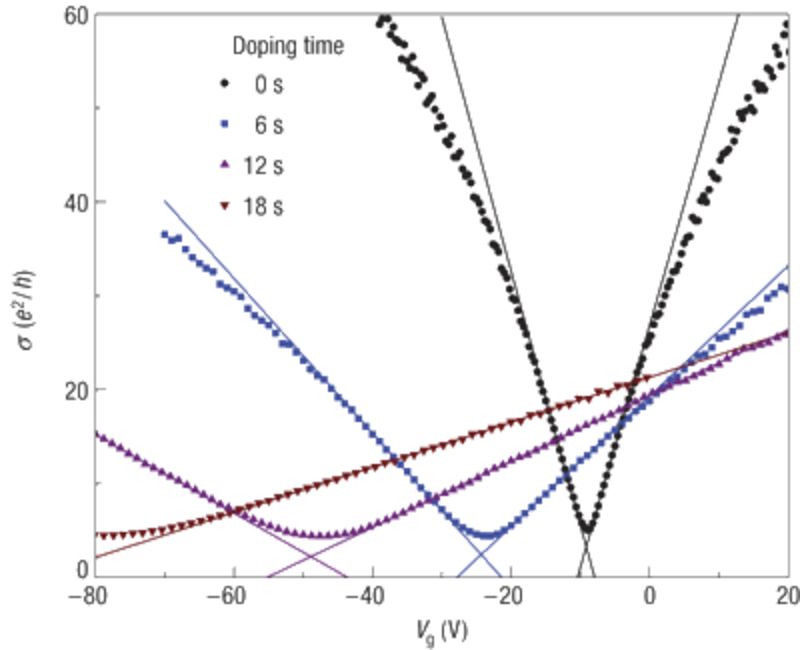
Conductivity as a function of the gate voltage (σ(*V*_g_)) of graphene at different exposures to K. The shift of neutrality point to negative gate voltages indicates an increase of n-type doping with increasing exposure to K atoms. The change in the slope of the curves suggest a reduction of the mobility of the charge carriers. Reprinted with permission from [29]. Copyright 2008 Nature Publishing Group.

**Figure 3:**
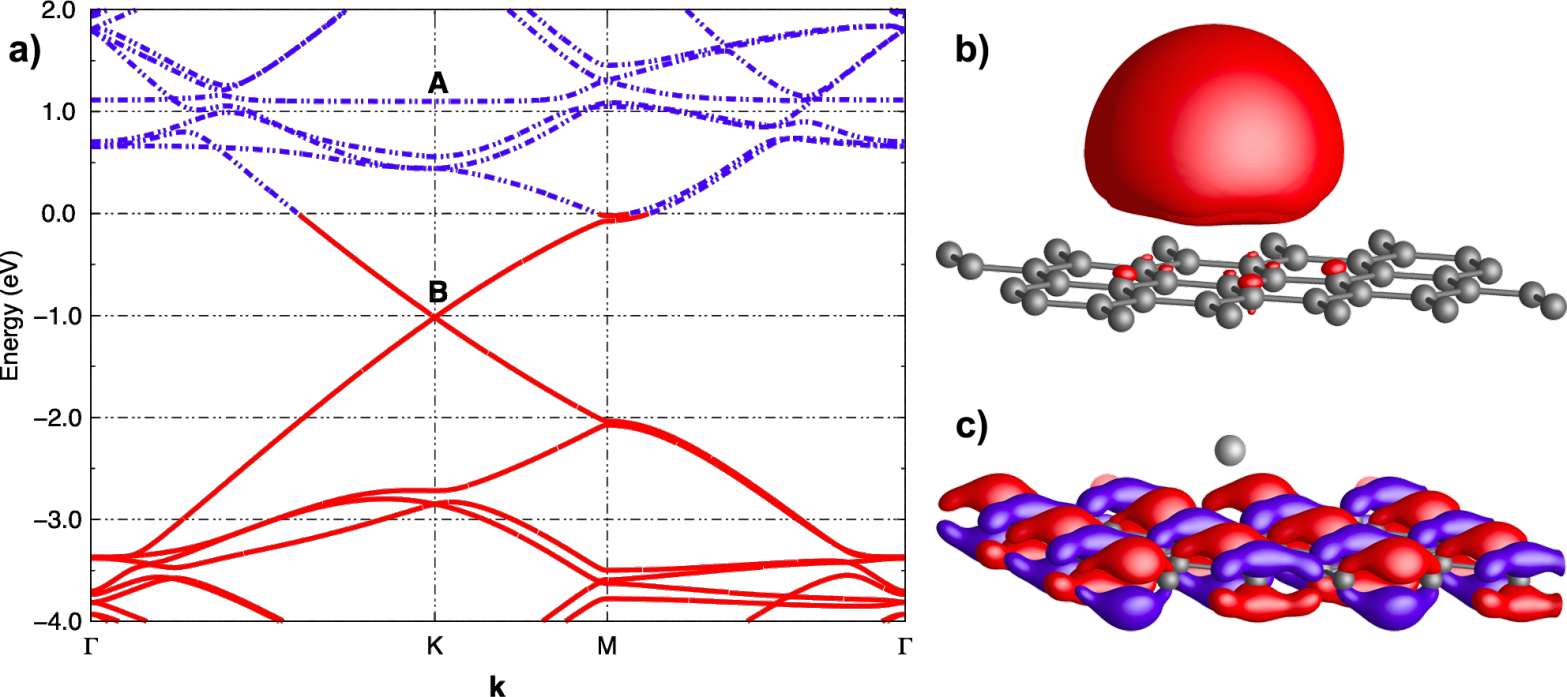
a) Electronic band structure (eV) of a K atom on top of a graphene layer in the vicinity of the Fermi energy. The Fermi level is set to zero. Full lines denote occupied states while dashed lines show empty levels. The bands around **B**, unoccupied for pristine graphene, are now occupied. b,c) Plots of the real part of the wavefunctions of electronic levels marked **A** and **B** in the electronic band structure of K on top of graphene. The wavefunction of the level marked **A** is localized on the K atom while the wavefunction of the highest occupied level, marked **B**, is delocalised over the graphene layer. These results confirm that charge transfer has occurred from the 4s occupied level of K to graphene. Reprinted with permission from [28]. Copyright 2010 American Physical Society.

The ability to dope graphene either n- or p-type has been demonstrated for various atomic and molecular adsorbates. Electropositive elements that easily donate their outer shell electrons are expected to be n-type dopants. Indeed, density functional theory (DFT) calculations predicted group I–III metals to be efficient electron donors for graphene [25–28]. Electron doping of graphene was observed experimentally for potassium (K) atoms (group I metal) deposited on graphene [29]. Figure 2 shows plots of the conductivity of graphene versus the gate voltage for different exposures to K. The shift of the neutrality point towards negative *V*_g_ with the increase of the potassium concentration is a clear indication of n-type doping. It was also observed that the mobility of the charge carriers decreases with the increase of the doping concentration. This is attributed to the additional scattering caused by the ionized potassium atoms. In agreement with the experimental results, DFT calculations have shown that K atoms act as electron donors [25–28]. Electronic band structure calculations show that adsorption of a K atom on graphene results in the shift of the Fermi level above the Dirac point, indicating the n-type doping of graphene, Figure 3a. Furthermore, it has been shown that the 4s^1^ electronic level of potassium, Figure 3b, which is occupied for an isolated K atom, becomes empty when the K atom is adsorbed on graphene (level marked A in Figure 3a). On the other hand, the wavefunctions of the occupied levels above the graphene Dirac point (marked B in Figure 3a) are delocalized over the carbon atoms of graphene (Figure 3c) indicating the presence of free electrons. These results confirm that n-type doping of graphene occurs due to the transfer of electrons from K atoms to graphene. Integration of the graphene density of states between the Dirac point and the Fermi level shows that approximately one electron is transferred per adsorbed K atom.

The doping properties were also explored for different transition metal clusters (Ti, Fe and Pt) deposited on graphene by molecular beam epitaxy (MBE) [30]. The Ti and Fe metal clusters were found to be n-type dopants with Ti being the most effective donor. Interestingly, deposition of Pt may lead to either n- or p-type doping depending on the Pt coverage. The n-type doping observed at low Pt-coverages is attributed to the formation of a strong interfacial dipole, causing a potential step which in this case promotes the n-type doping [31].

**Figure 4:**
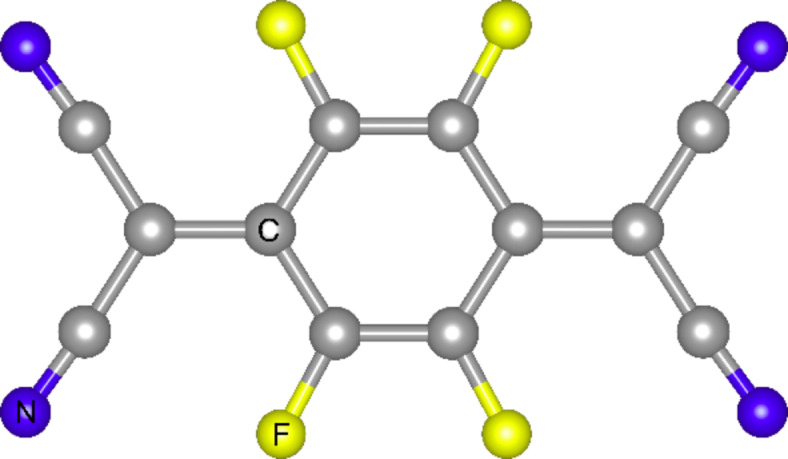
Molecular geometry of tetrafluorotetracyanoquinodimethane (F_4_-TCNQ).

Whereas one expects that electropositive adsorbates will dope graphene n-type, electronegative species should lead to p-type doping. By using angle-resolved photoemission spectroscopy Gierz et al. [32] demonstrated the doping of epitaxial graphene (EG) with holes by metal atoms with high electron affinity such as bismuth, antimony and gold. In the case of doping with bismuth and antimony the electron extraction from graphene only reduced the natural intrinsic n-type character of EG on SiC while gold actually shifted the Dirac point above the Fermi level, that is a clear evidence of induced p-type doping. Graphene layers with p-type conductivity can also be produced by deposition of some organic molecules. Tetrafluorotetracyanoquinodimethane (F_4_-TCNQ), shown in Figure 4, is an organic molecule with a strong electron affinity, which was previously demonstrated to be an efficient acceptor for diamond [33] and carbon nanotubes [34].

**Figure 5:**
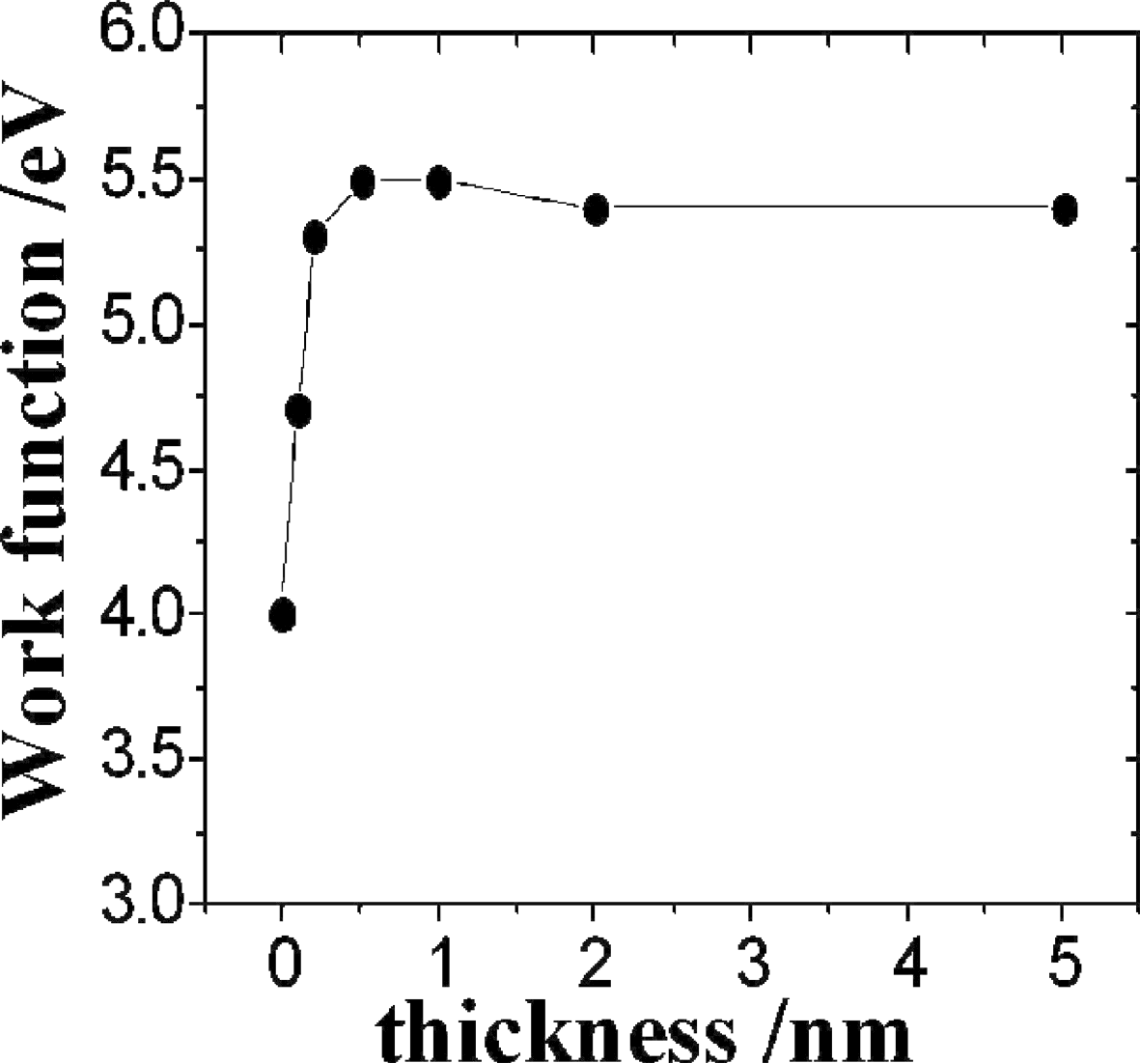
Plot of the workfunction of graphene (eV) as a function of the thickness (nm) of deposited F_4_-TCNQ. The workfunction of graphene increases 1.3 eV after the deposition of 0.2 nm of F_4_-TCNQ. For higher thicknesses the workfunction remains constant suggesting that the charge transfer occurs at the graphene/F_4_-TCNQ interface. Reprinted with permission from [35]. Copyright 2007 American Chemical Socieity.

As the workfunctions of diamond and graphene are similar, the F_4_-TCNQ molecule was considered to be a good candidate for p-type doping of graphene. The effect of the F_4_-TCNQ molecule on the electronic properties of epitaxial graphene was studied by synchrotron-based high resolution photoemission spectroscopy (PES) [35]. The increase of the graphene work function due to the deposition of F_4_-TCNQ (Figure 5) suggests that there is an electron transfer from graphene to the molecule. In agreement with PES measurements DFT calculations have shown the electron transfer of 0.3 *e* per molecule from the highest occupied electronic state of graphene to the lowest unoccupied electronic state of the F_4_-TCNQ molecule [36].

**Figure 6:**
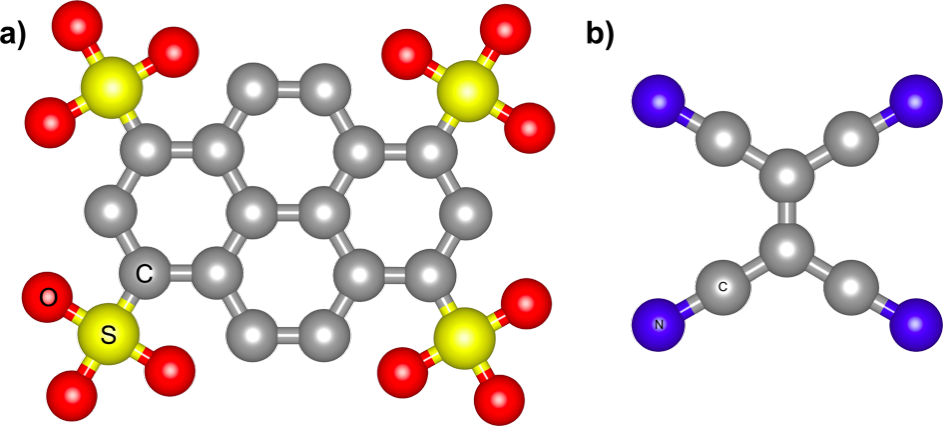
Molecular structure of a) pyrenetetrasulfonic acid (TPA) and b) tetracyanoethylene (TCNE).

The p-type doping of graphene can also be achieved by depositing other organic molecules such as pyrenetetrasulfonic acid (TPA) or tetracyanoethylene (TCNE) [37–38], shown in Figure 6a and Figure 6b, respectively. After the deposition of TPA, Raman spectroscopic studies showed upshifts of both Raman G and 2D frequencies compared to single layer graphene indicating p-type doping [37]. Electronic structure calculations based on density functional theory demonstrated that a p-type graphene can be obtained through charge transfer between the TCNE molecule and graphene [38].

### Electrochemical dopants

**Figure 7:**
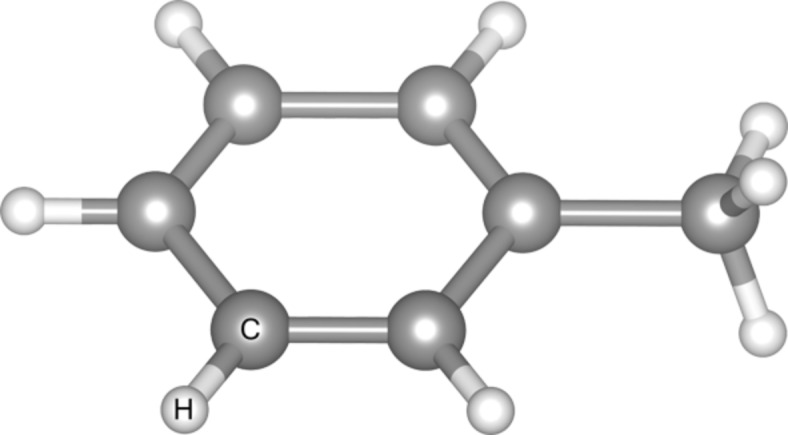
Molecular structure of toluene (C_6_H_5_CH_3_).

It was observed that the exposure of graphene to a humid atmosphere causes p-type doping whereas exposure to toluene (C_6_H_5_CH_3_), Figure 7, results in n-type doping [24,39–40]. However, doping of graphene in the presence of water and toluene cannot be understood within the electronic model. For instance, according to DFT calculations the Fermi level of graphene lies between the HOMO and LUMO levels of the adsorbed toluene molecules, which indicates the absence of electronic doping [41]. The doping effects in the above cases are instead attributed to electrochemical redox reactions occurring in aqueous layers at the graphene interface [41].

The *p*-doping induced by humid atmosphere occurs via a redox reaction involving H_2_O/O_2_ and graphene as in

[1]
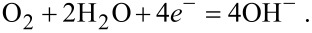


The value of Δ*G* of this reaction can be calculated from the tables of free energies to be −4.82 eV [19]. Under the assumption that electrons for the above reaction are taken from graphene the total Gibbs free energy change is −4.82 + *W* or −0.3 eV. Thus, the reaction will occur spontaneously near the graphene surface, making graphene p-type doped. The rate of the reaction is, however, influenced by the molar concentration of O_2_ and OH^−^. According to the Nernst equation Δ*G* can range from −4.8 to −5.7 eV in basic (pH 14) and acidic (pH 1) conditions, respectively. Therefore, the reaction is inhibited in basic conditions and promoted in acidic conditions since the concentration of OH^−^ is low.

The electrochemical p-type doping of graphene on a SiO_2_ substrate in the presence of O_2_ and H_2_O was confirmed experimentally by Raman spectroscopy measurements [39]. It was shown that the exposure of graphene to a mixture of O_2_ and H_2_O leads to a stronger doping than the exposure to O_2_ or H_2_O alone. The hysteresis effects commonly observed in graphene FETs doped under atmospheric conditions [21–24] were suppressed by treating the samples under vacuum for about 50 h or annealing at 473 K. The long evacuation time suggests that the doping species are strongly bound to graphene and difficult to be desorbed.

Similarly, the n-type doping of graphene caused by toluene can be explained by the electrochemical mechanism. In this case it is difficult to determine which particular reaction takes place. One possibility is the electrochemical oxidation of toluene to benzyl alcohol

[2]
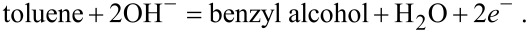


This reaction is spontaneous as the change of the total Gibbs free energy is −0.5 eV. The released electron can be trapped by graphene and make it n-type doped.

**Figure 8:**
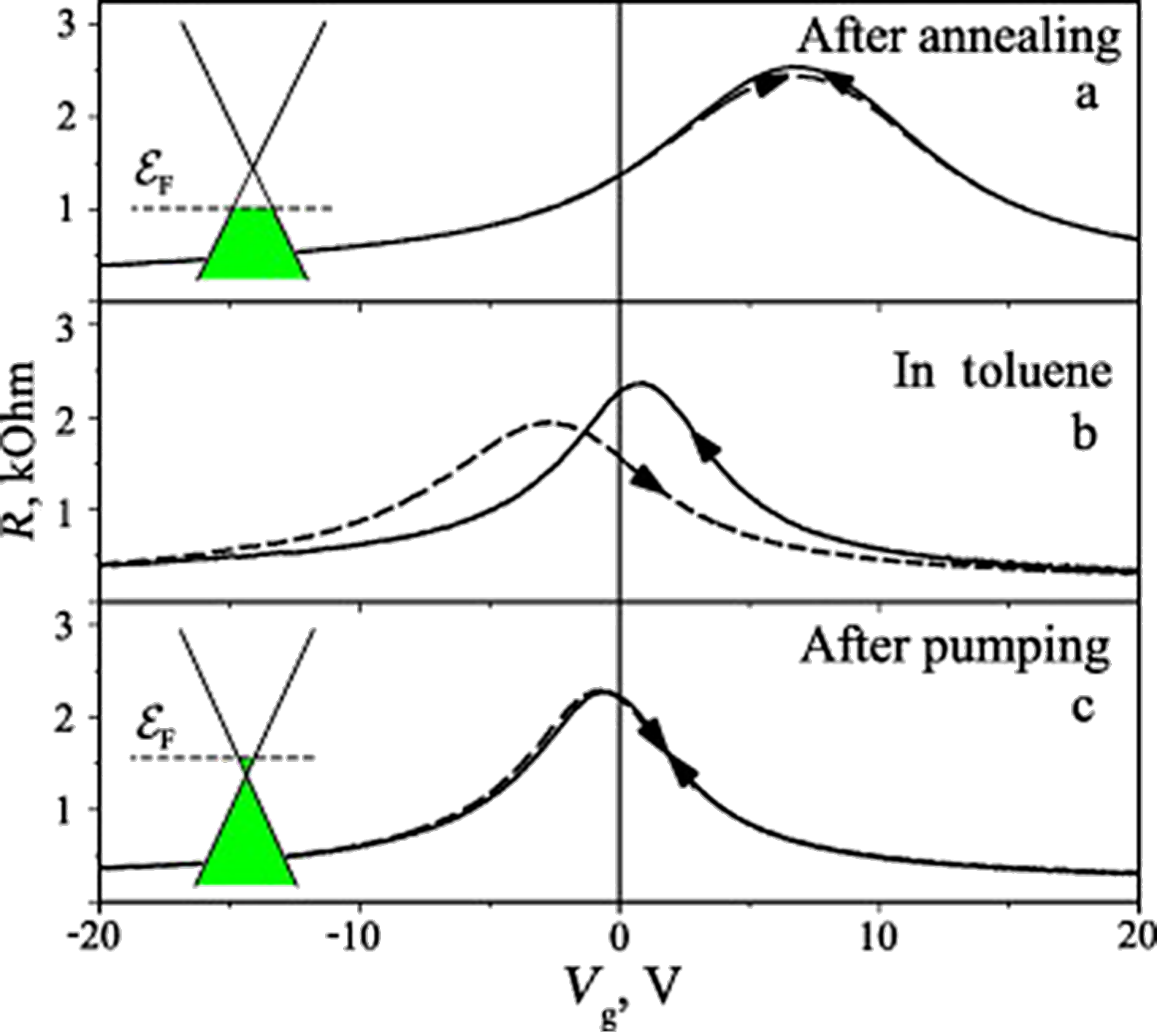
Resistance (kOhm) as a function of the gate voltage (*V*_g_) a) for pristine graphene after annealing in vacuum, b) after exposure to toluene and c) and pumping out the toluene vapour. The shift of the resistance peak towards negative (*V*_g_) after the exposure of toluene indicates that the molecule acts as a n-type dopant. Pumping out the toluene from the chamber removes the hysteresis but the doping effect remains. Reprinted with permission from [24]. Copyright 2011 Elsevier.

Figure 8 shows the measured resistance of a typical graphene FET device as a function of the gate voltage *V*_g_ at different stages of the toluene-doping experiment. Firstly, the sample was annealed in vacuum and the resistance peak at positive *V*_g_, Figure 8a, is an indication that the graphene is doped with holes. The exposure of graphene to toluene shifts the resistance peak to negative *V*_g_ showing that toluene acts as n-type dopant on graphene, Figure 8b. It is worth emphasizing that redox reactions are slow and thus graphene needs to be exposed to toluene for long periods of about 1 h for a doping effect to be seen. Furthermore, hysteresis is observed in the *R*(*V*_g_) measurement. The position of the resistance peak changes with the direction of the *V*_g_ sweep. The hysteresis vanished when the toluene is pumped out, but the doping effect remains, Figure 8c.

Hysteresis effects are often observed in graphene FETs based on Si^+^/SiO_2_ substrates [21–24]. Such effects are not expected in the case of electronic doping. However, they can be explained within the electrochemical doping model if a chemical reaction rate or diffusion rates of species involved in the reaction are slower than the rate of gate voltage change. The most obvious reaction in this case is the reaction in Equation 1. The water molecules required for the reaction are assumed to be located at the graphene/SiO_2_ interface. Indeed, the presence of water layers on the surface of SiO_2_ films grown on Si has been confirmed by Kelvin probe microscopy, X-ray spectroscopy and FTIR measurements [42–43]. Water molecules are also believed to be present in small voids in amorphous SiO_2_. It has been found that for graphene FETs operating under ambient conditions the hysteresis is suppressed when Si^+^/SiO_2_ substrate was covered with a thin hydrophobic layer [23]. Another experiment showed that atmospheric doping does not occur in free standing graphene [39], supporting that the main source of water molecules is within the substrate or at the graphene/SiO_2_ interface. The binding energy of OH^−^ to the SiO_2_ network was estimated to be about 0.3 eV indicating that OH^−^ can easily diffuse through SiO_2_ at room temperature [44]. Therefore, the concentration of OH^−^ at the graphene/SiO_2_ interface and thus the rate of the reaction is influenced by the gate voltage. A positive *V*_g_ results in the reduction of the OH^−^ concentration at the graphene interface promoting the further dissociation of H_2_O molecules, which consequently increases the concentration of holes in graphene, as it was observed experimentally [21].

Some of these redox reactions were first discussed for diamond and carbon nanotubes [17,45] and were investigated for graphene because of the similarity in all their work functions. For instance, the exposure of hydrogenated diamond to a humid atmosphere results in p-type doping [46–47], which can be suppressed by NH_3_ and enhanced by NO_2_ [48]. Similar effects were reported for carbon nanotubes [49–50]. It would be interesting to see whether they could apply to graphite or indeed if some of the variant reactions tried on graphene could be applied to diamond, graphite and carbon nanotubes.

## Conclusion

In this article we have revisited the surface dopants of graphene and the charge transfer mechanisms. The deposition of atoms or molecules is an effective method to control the type and concentration of charge carriers in graphene. Surface charge transfer can be mediated by two mechanisms, electronic and electrochemical doping. The electronic doping occurs by direct exchange of electrons between graphene and the adsorbates, either atoms or molecules. Electropositive elements that easily donate their outer shell electrons, such as potassium, are expected to be n-type dopants while adsorbates with a strong electron affinity, such as F_4_-TCNQ, have been proved to be very efficient p-type dopants. This type of doping occurs instantaneously and should not cause hysteresis effects. However, the ionized dopants become additional charge scatterers leading to a reduction in the charge carrier mobility.

The electrochemical doping of graphene occurs as a result of redox reactions that can take place near the graphene surface. Unlike electronic doping it does not reduce the charge carriers mobility but it may require appreciable time to occur since the reaction and diffusion barriers have to be overcome. Moreover, the electrochemical doping model can explain the hysteresis effects usually observed in graphene based field effect transistors when operating in atmospheric environment.

## References

[1] Novoselov K S, Geim A K, Morozov S V, Jiang D, Zhang Y, Dubonos S V, Grigorieva I V, Firsov A A (2004). Science.

[2] Novoselov K S, Fal’ko V I, Colombo L, Gellert P R, Schwab M G, Kim K (2012). Nature.

[3] Geim A K, Novoselov K S (2007). Nat Mater.

[4] Novoselov K S, Geim A K, Morozov S V, Jiang D, Katsnelson M I, Grigorieva I V, Dubonos S V, Firsov A A (2005). Nature.

[5] Nair R R, Blake P, Grigorenko A N, Novoselov K S, Booth T J, Stauber T, Peres N M R, Geim A K (2008). Science.

[6] Lee C, Wei X, Kysar J W, Hone J (2008). Science.

[7] Bae S, Kim H, Lee Y, Xu X, Park J-S, Zheng Y, Balakrishnan J, Lei T, Kim H R, Song Y I (2010). Nat Nanotechnol.

[8] Li X, Zhu Y, Cai W, Borysiak M, Han B, Chen D, Piner R D, Colombo L, Ruoff R S (2009). Nano Lett.

[9] Xia F, Mueller T, Golizadeh-Mojarad R, Freitag M, Lin Y-m, Tsang J, Perebeinos V, Avouris P (2009). Nano Lett.

[10] Lin Y-M, Dimitrakopoulos C, Jenkins K A, Farmer D B, Chiu H-Y, Grill A, Avouris P (2010). Science.

[11] Wallace P R (1947). Phys Rev.

[12] Castro Neto A H, Guinea F, Peres N M R, Novoselov K S, Geim A K (2009). Rev Mod Phys.

[13] Reddy D, Register L F, Carpenter G D, Banerjee S K (2011). J Phys D: Appl Phys.

[14] Liu H, Liu Y, Zhu D (2011). J Mater Chem.

[15] Panchakarla L S, Subrahmanyam K S, Saha S K, Govindaraj A, Krishnamurthy H R, Waghmare U V, Rao C N R (2009). Adv Mater.

[16] Wei D, Liu Y, Wang Y, Zhang H, Huang L, Yu G (2009). Nano Lett.

[17] Hansen W N, Hansen G J (2001). Surf Sci.

[18] Sque S J, Jones R, Briddon P R (2007). Phys Status Solidi A.

[19] Lide D R (2005). CRC Handbook of Physics and Chemistry.

[20] Reiss H (1985). J Phys Chem.

[21] Lohmann T, von Klitzing K, Smet J H (2009). Nano Lett.

[22] Xu H, Chen Y, Xu W, Zhang H, Kong J, Dresselhaus M S, Zhang J (2011). Small.

[23] Lafkioti M, Krauss B, Lohmann T, Zschieschang U, Klauk H, von Klitzing K, Smet J H (2010). Nano Lett.

[24] Kaverzin A A, Strawbridge S M, Price A S, Withers F, Savchenko A K, Horsell D W (2011). Carbon.

[25] Chan K T, Neaton J B, Cohen M L (2008). Phys Rev B.

[26] Liu X, Wang C Z, Yao Y X, Lu W C, Hupalo M, Tringides M C, Ho K M (2011). Phys Rev B.

[27] Uchoa B, Lin C-Y, Castro Neto A H (2008). Phys Rev B.

[28] Pinto H, Jones R, Goss J P, Briddon P R (2010). Phys Rev B.

[29] Chen J-H, Jang C, Adam S, Fuhrer M S, Williams E D, Ishigami M (2008). Nat Phys.

[30] Pi K, McCreary K M, Bao W, Han W, Chiang Y F, Li Y, Tsai S-W, Lau C N, Kawakami R K (2009). Phys Rev B.

[31] Giovannetti G, Khomyakov P A, Brocks G, Karpan V M, van den Brink J, Kelly P J (2008). Phys Rev Lett.

[32] Gierz I, Riedl C, Starke U, Ast C R, Kern K (2008). Nano Lett.

[33] Qi D, Chen W, Gao X, Wang L, Chen S, Loh K P, Wee A T S (2007). J Am Chem Soc.

[34] Nosho Y, Ohno Y, Kishimoto S, Mizutani T (2007). Nanotechnology.

[35] Chen W, Chen S, Qi D C, Gao X Y, Wee A T S (2007). J Am Chem Soc.

[36] Pinto H, Jones R, Goss J P, Briddon P R (2009). J Phys: Condens Matter.

[37] Dong X, Fu D, Fang W, Shi Y, Chen P, Li L-J (2009). Small.

[38] Lu Y H, Chen W, Feng Y P, He P M (2009). J Phys Chem B.

[39] Xu H, Chen Y, Zhang J, Zhang H (2012). Small.

[40] Schedin F, Geim A K, Morozov S V, Hill E W, Blake P, Katsnelson M I, Novoselov K S (2007). Nat Mater.

[41] Pinto H, Jones R, Goss J P, Briddon P R (2010). Phys Status Solidi A.

[42] Verdaguer A, Weis C, Oncins G, Ketteler G, Bluhm H, Salmeron M (2007). Langmuir.

[43] Aarts I M P, Pipino A C R, Hoefnagels J P M, Kessels W M M, van de Sanden M C M (2005). Phys Rev Lett.

[44] Bakos T, Rashkeev S N, Pantelides S T (2004). Phys Rev B.

[45] Rezek B, Sauerer C, Nebel C E, Stutzmann M, Ristein J, Ley L, Snidero E, Bergonzo P (2003). Appl Phys Lett.

[46] Maier F, Riedel M, Mantel B, Ristein J, Ley L (2000). Phys Rev Lett.

[47] Chakrapani V, Angus J C, Anderson A B, Wolter S D, Stoner B R, Sumanasekera G U (2007). Science.

[48] Garrido J A, Härtl A, Dankerl M, Reitinger A, Eickhoff M, Helwig A, Müller G, Stutzmann M (2008). J Am Chem Soc.

[49] Kong J, Franklin N R, Zhou C, Chapline M G, Peng S, Cho K, Dai H (2000). Science.

[50] Bradley K, Gabriel J-C P, Briman M, Star A, Grüner G (2003). Phys Rev Lett.

